# CO_2_ Absorption Mechanism by the Nonaqueous Solvent Consisting of Hindered Amine 2-[(1,1-dimethylethyl)amino]ethanol and Ethylene Glycol

**DOI:** 10.3390/molecules25235743

**Published:** 2020-12-05

**Authors:** Ran Li, Congyi Wu, Dezhong Yang

**Affiliations:** 1 School of Earth Sciences and Resources, China University of Geosciences, Beijing 100083, China; ran594244379@163.com; 2 School of Science, China University of Geosciences, Beijing 100083, China; wucongyi@cugb.edu.cn

**Keywords:** capture, carbon dioxide, greenhouse gas, hindered amine, water lean

## Abstract

In this work, we studied the CO_2_ absorption mechanism by nonaqueous solvent comprising hindered amine 2-[(1,1-dimethylethyl)amino]ethanol (TBAE) and ethylene glycol (EG). The NMR and FTIR results indicated that CO_2_ reacted with an -OH group of EG rather than the -OH of TBAE by producing hydroxyethyl carbonate species. A possible reaction pathway was suggested, which involves two steps. In the first step, the acid–base reaction between TBAE and EG generated the anion HO-CH_2_-CH_2_-O-; in the second step, the O^−^ of HO-CH_2_-CH_2_-O^−^ attacked the C atom of CO_2_, forming carbonate species.

## 1. Introduction

In recent decades, the amount of carbon dioxide (CO_2_), the main heat-trapping gas, accumulated in the air, has reached an unbelievable level, which is viewed as the main contributor to global warming causing severe environmental problems such as the rising atmospheric temperature, intense heat waves and drought. The vast majority of atmospheric CO_2_ is mainly emitted from industrial activities by burning fossil fuels such as coal and oil to produce electricity [1]. An urgent demand to curb the atmospheric CO_2_ concentration to avoid a climate disaster has driven industry and the scientific community to explore efficient CO_2_ capture technologies. A current popularly used method for CO_2_ capture in industry is the amine-based scrubbing process, which mainly utilizes the aqueous solution of alkanolamine to chemically absorb CO_2_ [2]. However, amine-based sorption systems have several drawbacks, such as high solvent volatility and equipment corrosion as well as high energy penalty of absorbent regeneration [3,4]. To develop new and efficient sorption systems capable of addressing the above-mentioned drawbacks is one of the main challenges in the field of carbon capture and storage.

In recent years, nonaqueous solvent blends formed by amine and a conventional organic solvent have been developed as promising alternatives to the aqueous amine absorbents, because they can provide the advantage of reducing the energy cost during the regeneration steps while retaining high CO_2_ capacity [5]. At present, many nonaqueous systems were studied to capture CO_2_, such as the mixture of alcohol and amidines [6], alkanolamine solutions such as monoethanolamine (MEA) and 2-amino-2-methyl-1-propanol (AMP) in ethanol or ethylene glycol (EG) [7,8,9,10,11,12,13,14], and the amine-based deep eutectic solvents [15,16,17].

**Scheme 1:**
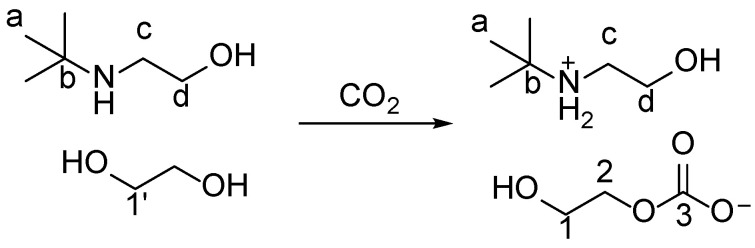
The reaction of CO_2_ with amine 2-[(1,1-dimethylethyl)amino]ethanol (TBAE) and ethylene glycol (EG).

Im and co-authors reported that the sterically hindered amine 2-[(1,1-dimethylethyl)amino]ethanol (TBAE) in EG solution could capture CO_2_ by forming zwitterionic carbonate species, and they believe that CO_2_ reacted with the -OH group of TBAE rather than reacted with the -OH group of EG [18]. However, Xie and co-authors reported that CO_2_ reacted with EG by forming hydroxyethyl carbonate species and the zwitterionic carbonate species produced from the reaction between CO_2_ and TBAE was not the main product when CO_2_ was captured by the solution of TBAE in EG, which is supported by quantum chemical calculations and mass spectrometry [19]. In other words, the results reported by these two groups contradicted each other. Therefore, in this work, in order to clarify the reaction between CO_2_ and TBAE-EG absorbent, we studied the CO_2_ absorption mechanism again using Nuclear Magnetic Resonance (NMR) and Fourier Transform Infrared Spectrometer (FTIR) spectroscopies. Our results suggested that CO_2_ reacted with the -OH group of EG with the formation of a carbonate species (Scheme 1), not with the -OH group of TBAE, which was consistent with the results reported by Xie [19]. The details of our investigation can be found in the following section.

## 2. Results and Discussions

**Figure 1:**
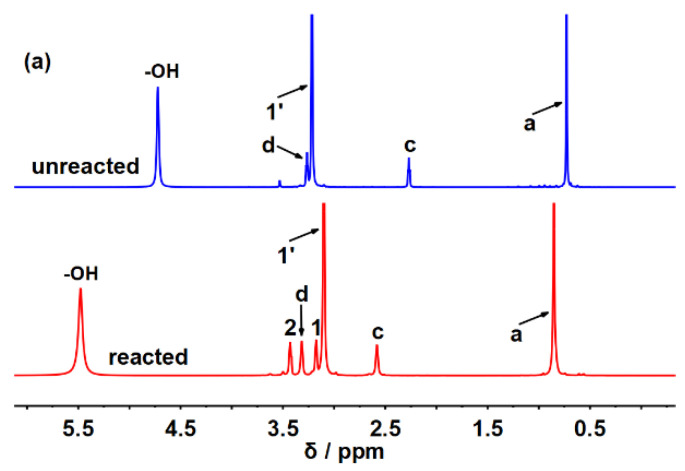

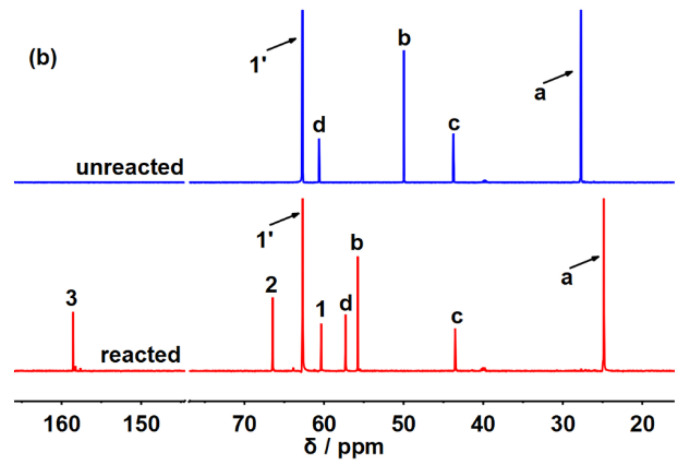
NMR spectra of TBAE-EG system before and after CO_2_ absorption: (**a**) ^1^H NMR; (**b**) ^13^C NMR.

The interaction between CO_2_ and the TBAE-EG system are characterized using NMR and FTIR spectra. The ^1^H and ^13^C NMR spectra of TBAE-EG system before and after CO_2_ absorption can be seen in Figure 1. As shown in Figure 1a, two new peaks at 3.17 (H-1) and 3.43 (H-2) ppm can be found in the ^1^H NMR spectrum of TBAE-EG after absorption, and three new peaks appeared at 60.4 (C-1), 66.5 (C-2) and 158.6 (C-3) ppm in the ^13^C NMR spectrum after absorption (Figure 1b). The new peaks in the NMR spectra cannot be explained if CO_2_ reacted with the -OH group of TBAE to form zwitterionic carbonate species.

**Figure 2:**
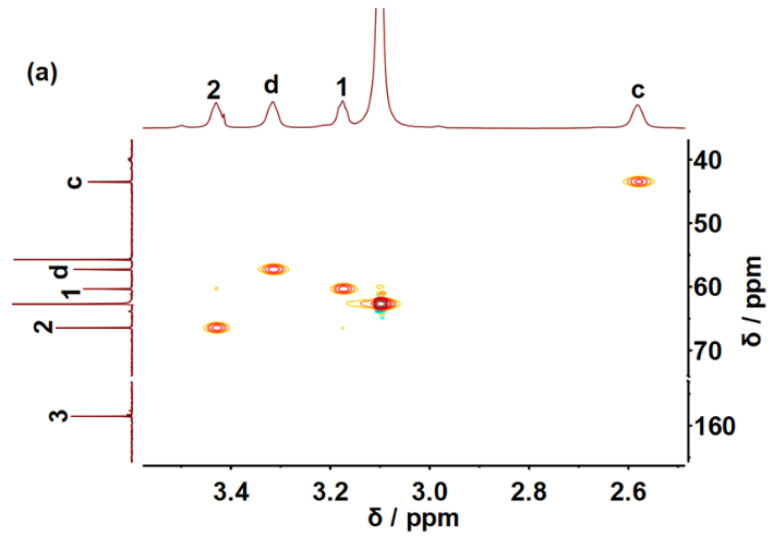

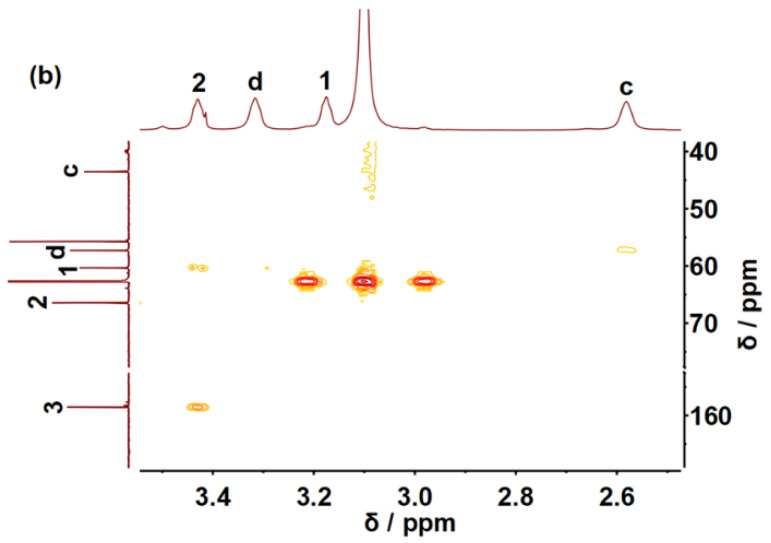
The 2D NMR spectra of TBAE-EG after CO_2_ absorption: (**a**) ^1^H-^13^C Heteronuclear Single Quantum Coherence (^1^H-^13^C HSQC); (**b**) ^1^H-^13^C Heteronuclear Multiple Bond Correlation (^1^H-^13^C HMBC).

In order to explain the new peaks in the NMR spectra, the 2D NMR spectra of ^1^H-^13^C Heteronuclear Single Quantum Coherence (HSQC) and ^1^H-^13^C Heteronuclear Multiple Bond Correlation (HMBC) of the TBAE-EG system after CO_2_ uptake were studied. As shown in ^1^H-^13^C HSQC spectroscopy (Figure 2a), H-1 and H-2 correlated with C-1 and C-2, respectively. In the ^1^H-^13^C HMBC spectroscopy (Figure 2b), the C-3 carbon did not correlate with the H-d hydrogen of TBAE, indicating that CO_2_ did not react with the -OH group of TBAE, and there was also no correlation between the C-3 carbon and the H-c hydrogen of TBAE, indicating CO_2_ was not attached the amine group of TBAE. The HMBC results revealed that CO_2_ did not directly react with TBAE. Therefore, CO_2_ should react with EG, the other species in the TEAE-EG system, which was supported by the HMBC results. As presented in Figure 2b, the C-3 carbon correlated with the H-2 hydrogen and the C-1 carbon also correlated with the H-2 hydrogen, suggesting that CO_2_ directly reacted with the -OH group of EG by forming a carbonate species. The peaks of C-1 and C-2 can be ascribed to the carbon atoms of the carbonate species derived from EG, which were similar to those found in the AMP-EG-based nonaqueous solution after carbon capture [9]. The C-3 carbon was the carbonyl carbon in the EG-derived carbonate species [20]. Moreover, the peak of C-b carbon attached to the N atom of TBAE shifted downfield from 50.0 to 55.8 ppm after CO_2_ absorption, which indicates that the amine group of TBAE also plays a role in CO_2_ capture. On the basis of the above results, it can be concluded that CO_2_ reacted with the -OH of EG by producing carbonate species and the amine group of TBAE obtains a proton after CO_2_ uptake.

**Figure 3:**
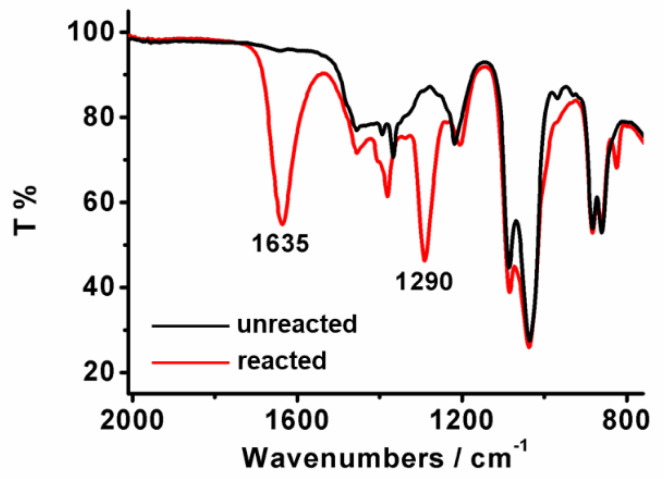
The FTIR spectra of TBAE-EG before and after CO_2_ absorption.

The FTIR spectra of the TBAE-EG system with and without CO_2_ were also studied. Two new peaks can be observed at 1635 and 1290 cm^−1^ (Figure 3), which can be ascribed to the asymmetric and symmetric carbonyl stretching frequency of C=O in R-O-COO^−^ species [6]. However, the two peaks were different from those of TBAE-CO_2_ adduct (1641 and 1295 cm^−1^) reported by Im and co-authors [18], suggesting that CO_2_ was bonded to the O atom of EG, not the O atom of TBAE. Therefore, the FTIR results confirmed again the reaction between CO_2_ and -OH of EG.

**Scheme 2:**
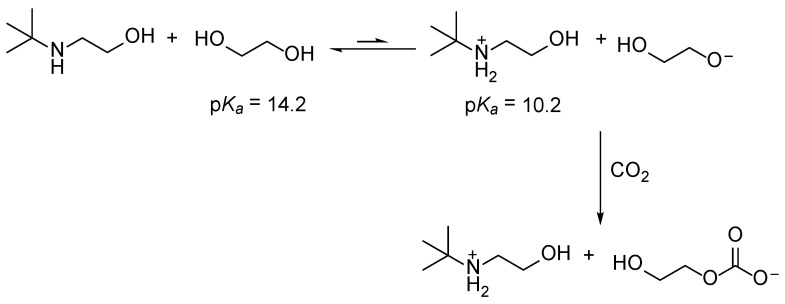
The possible reaction pathway for the reaction between CO_2_ and TBAE-EG.

On the basis of the above discussion, we think that the reaction pathway involves two steps (Scheme 2). At first, there was an acid–base reaction between TBAE and EG, forming the anion HO-CH_2_-CH_2_-O^−^, the conjugate base of EG. The equilibrium constant (*K*_eq_) of the acid-base reaction can be obtained using the following equations: (see [18,21]).

p*K_eq_* = p*K_a_* (EG) − p*K_a_* ([TBAEH]^+^) = 4.0. (1)

*K*_eq_ = 1.0 × 10^−4^ (2)

In the second step, the anion HO-CH_2_-CH_2_-O^−^ reacted with CO_2_ to form the carbonate species.

It is reasonable to anticipate that the proton of the OH group of TBAE can transfer to the amino group by forming zwitterionic species (CH_3_)_3_-C-N^+^(H_2_)-CH_2_-CH_2_-O^−^ in TBAE-EG absorbent, which may react with CO_2_ to form zwitterionic carbonates. However, NMR results did not show any signals of zwitterionic carbonates, suggesting that the reaction pathway forming zwitterionic carbonates was not preferable in the TBAE-EG system. In order to further confirm the reaction between CO_2_ and EG, we studied the reaction between CO_2_ and a nonaqueous solvent consisting of superbase 1,5-diazabicyclo [5.4.0]-5-undecene (DBU) and EG. As reported in the literature, CO_2_ reacted with the -OH group of alcohol when CO_2_ was absorbed by the DBU–alcohol mixtures [22]. The ^1^H and ^13^C NMR spectra of DBU solution (30 wt%) in EG before and after CO_2_ absorption were shown in Figure S1. There were two new peaks at 3.15 and 3.39 ppm in the ^1^H NMR spectra of DBU-EG after absorption (Figure S1a). Three new peaks appeared at 60.0 (C-1), 65.8 (C-2) and 157.9 (C-3) ppm in the ^13^C NMR after absorption (Figure S1b). These new peaks in the DBU-EG-CO_2_ system were consistent with those in the TBAE-EG-CO_2_ system, suggesting that CO_2_ reacted with EG. These results again suggested that the CO_2_ absorption mechanism of TBAE-EG presented in this work was understandable.

The desorption of CO_2_ was also investigated. CO_2_ captured by TBAE-EG can be desorbed at 80 °C and the results were characterized by NMR and FTIR. As shown in the ^1^H and ^13^C NMR spectra of TBAE-EG after CO_2_ desorption (Figure S2), the peaks of carbonate anions cannot be observed, suggesting CO_2_ was released after heating. The asymmetric and symmetric carbonyl stretching frequencies clearly disappeared in the FTIR spectra (Figure S3) after CO_2_ desorption, indicating again the reaction between CO_2_ and TBAE-EG was reversible. The results reported by Im and co-authors [18] also showed the good reversibility of TBAE-EG solvent for CO_2_ capture.

## 3. Conclusions

The results indicated that CO_2_ reacted with -OH group of EG in nonaqueous solvent consisting of TBAE and EG rather than reacting with -OH group of TBAE when CO_2_ was captured by the mixture consisting of TBAE and EG. We believe the confirmation of the absorption mechanism is very important to the design of new nonaqueous absorption systems in the future.

## 4. Experimental Sections

### 4.1. Materials and Characterization

TBAE (98%) and EG (99.5%) were purchased from J&K Scientific Ltd (Beijing, China). CO_2_ (≥99.99%) and N_2_ (≥99.99%) was supplied by Beijing ZG Special Gases Sci. and Tech. Co. Ltd (Beijing, China). DBU (98%) was obtained from Alfa Aesar (Shanghai, China).

The FTIR spectra were recorded on a PerkinElmer Frontier spectrometer (PerkinElmer Corp., Waltham, MA, USA). ^1^H NMR (600 MHz) and ^13^C NMR (151 MHz) data were obtained from a Bruker spectrometer (Bruker Biospin, Karlsruhe, Germany) and DMSO-d_6_ was used as the external reference. The NMR spectrometer was equipped with a 5 mm PABBO probe. The experiment temperature was 25 °C. For the ^1^H NMR experiment, the relaxation delay was 1.0 s, and the acquisition time was 2.75 s. In the ^13^C NMR experiment, the relaxation delay was 2.0 s, and the acquisition time was 0.92 s. For the HMBC experiment, the relaxation delay was 1.5 s, and the acquisition time was 0.18 s. For the HSQC experiment, the relaxation delay was 1.5 s, and the acquisition time was 0.18 s.

### 4.2. Absorption of CO_2_

The method of CO_2_ absorption was similar to that reported by Im and co-authors. TBAE solution (30 wt%) in EG (~2 g) was added into a glass tube with a diameter of 10 mm equipped with a rubber lid. At first, the tube was partially placed into an oil bath at 100 °C and N_2_ (50 mL/min) was bubbled into the solution through a needle for 10 min to remove any volatile compounds. Then, CO_2_ (50 mL/min) was bubbled into the solution in the tube at 40 °C for 60 min, and the weight of the tube during absorption was recorded using a balance (±0.1 mg).

## Supplementary Materials

The following are available online. Figure S1: The ^1^H (**a**) and ^13^C NMR (**b**) spectra of DBU-EG before and after CO_2_ absorption, Figure S2: The ^1^H (**a**) and ^13^C NMR (**b**) spectra of TBAE-EG after CO_2_ desorption, Figure S3: The FTIR spectra of TBAE-EG after CO_2_ desorption.
