# Supplementary material for: CO2 Absorption Mechanism by the Nonaqueous Solvent Consisting of Hindered Amine 2-[(1,1-dimethylethyl)amino]ethanol and Ethylene Glycol
Source: Molecules. 2020 Dec 5;25(23):5743. doi: 10.3390/molecules25235743 (PMC7729447; doi:10.3390/molecules25235743)
Supplement: Supplementary file 1 [file molecules-25-05743-s001.pdf]

Supplementary

CO_2 Absorption Mechanism by the Nonaqueous Solvent Consisting of Hindered Amine 2-[(1,1-dimethylethyl)amino]ethanol and Ethylene Glycol

Ran Li [1], Congyi Wu [2] and Dezhong Yang [2,*]

[1] School of Earth Sciences and Resources, China University of Geosciences, Beijing 100083, China; ran594244379@163.com

[2] School of Science, China University of Geosciences, Beijing, 100083, China; wucongyi@cugb.edu.cn

* Correspondence: yangdz@cugb.edu.cn

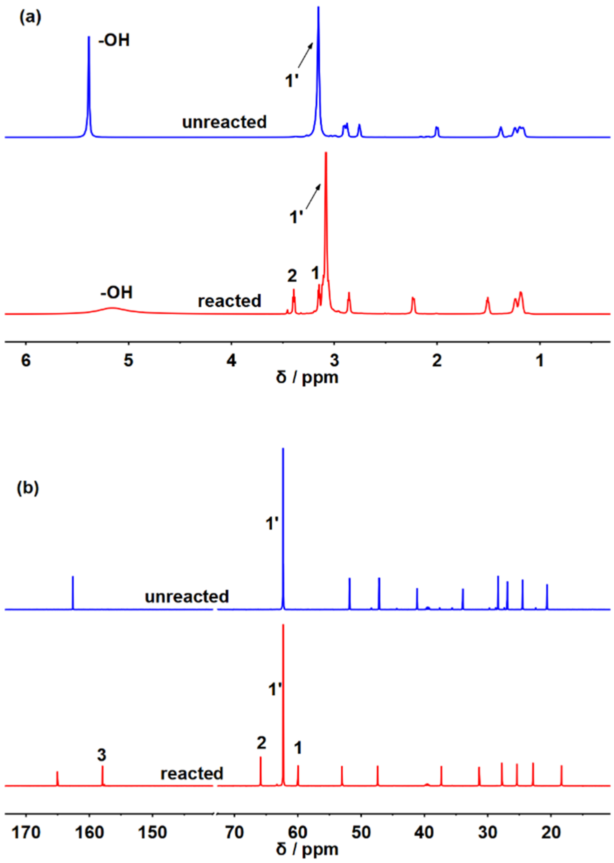

Figure S1. The ^{1}H (**a**) and ^{13}C NMR (**b**) spectra of DBU-EG before and after CO_2 absorption.

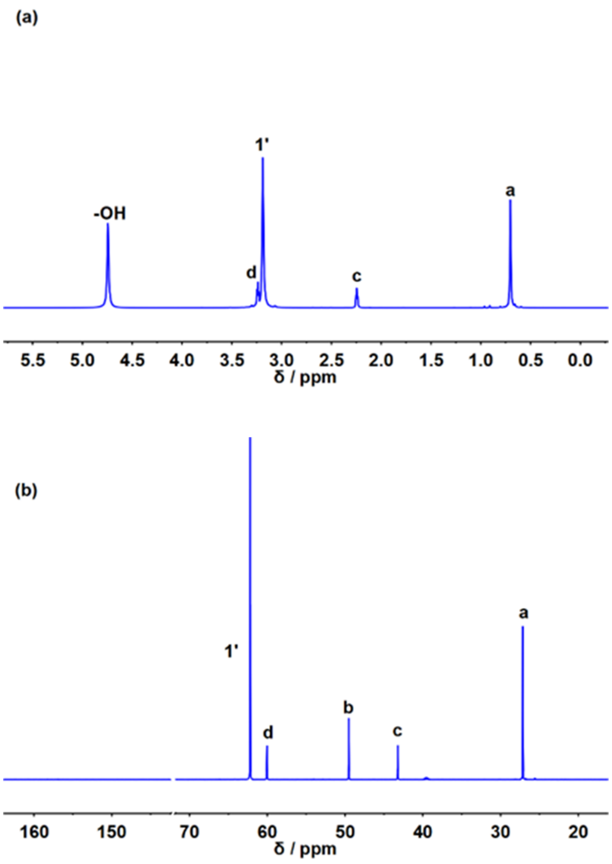

Figure S2. The ^{1}H (**a**) and ^{13}C NMR (**b**) spectra of TBAE-EG after CO_2 desorption.

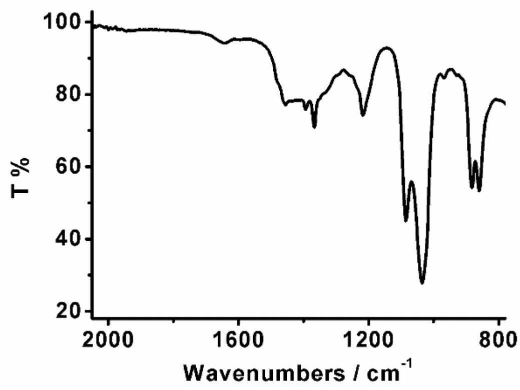

Figure S3. The FTIR spectra of TBAE-EG after CO_2 desorption.
